# Supplementary material for: Comparative Analysis of Five Multiplex RT-PCR Assays in the Screening of SARS-CoV-2 Variants
Source: Microorganisms. 2022 Jan 27;10(2):306. doi: 10.3390/microorganisms10020306 (PMC8876857; doi:10.3390/microorganisms10020306)
Supplement: Supplementary file 1 [file microorganisms-10-00306-s001.zip › microorganisms-1551040-supplementary.pdf]

Table S1. Analysis and interpretation of melting peaks results for L452 and E484 mutations (UltraGene Assay SARS-CoV-2 452R & 484K & 484Q Mutations V1).

	**Melting peak (C°)**	**Analysis and interpretation**
Mutation L452R	>65	L452R
	61-65	L452 Wild Type
	<61	Other L452 mutation
	No peaks	Uninterpretable
Mutation E484	>63	E484K
	59-63	E484Q
	55-59	E484E (wild type)
	<55	Other E484 mutation
	No peaks	Uninterpretable

Melting temperature (Tm) ranges for 452 and 484 positions are calculated using CFX96 instrument thermal cycler of seven reference controls harboring or not mutations L452R, E484K o E484Q. Samples with melting temperature < 61°C are characterized by L452Q mutation as confirmed by whole genome sequencing. The identification of Delta variant changes the Tm of E484 target that it decreases from 57°C to 56°C for the presence of T478K mutation.

Table S2. Analysis of results through temperature melting ranges for L452, E484 and N501Y mutations (SARS-CoV-2 Extended ELITe MGB).

	**Temperature melting range (C°)**	**Result**
Mutation L452R	54-61	L452R
	61.1-68	L452R NOT detected
Mutation E484	59.5-63.9	E484K
	56-59.4	E484Q
	64-70	E484K/Q NOT detected
Mutation N501Y	61-67	N501Y
	54-60.9	N501Y NOT detected

Internal Control endogenous is amplified for each sample. All steps are automatically performed on ELITe InGenius instrument using FAM, AP593, AP639 annd AP525 for the 484, 501, 452 and internal control respectively. Temperature melting ™ values of the wild type and positive controls must be interpretated together to the Tm of negative control according to the manufacturer instructions.

Table S3. Reference melting temperature ranges for the interpretation of the L452R, E484 and N501Y mutation of samples tested (Simplexa SARS-CoV-2 Variants Direct).

	**Melting temperature (C°)**	**Variant interpretation**
Mutation L452R	54.3-57.9	L452R
	48.7-52.1	L452 NOT detected
Mutation E484	51.2-54.6	E484K
	46.7-51.1	E484Q
	43.8-46.6	E484 NOT detected
Mutation N501Y	64.8-69.0	N501Y
	60.9-64.7	N501Y NOT detected

Detection of the Simplexa RNA Internal Control is not nequired for a valid result. The fluorescent signal for each specific probe is read using CFX96 instrument thermal cycler through channel 610 for L452R mutation; channel 560 for E484 mutation and channel 520 for N501Y mutation. Tm values of the wild type samples are the same of the positive control of reference.
